# Dynamic alterations of plasma cell free DNA in response to chemotherapy in children with neuroblastoma

**DOI:** 10.1002/cam4.2045

**Published:** 2019-02-21

**Authors:** Yan Su, Lijun Wang, Xisi Wang, Zhixia Yue, Tianyu Xing, Wen Zhao, Qian Zhao, Chao Duan, Cheng Huang, Yi Han, Lihua Qiu, Xianfeng Cheng, Yi Liu, Xiaoli Ma

**Affiliations:** ^1^ Beijing Key Laboratory of Pediatric Hematology Oncology, National Discipline of Pediatrics, Ministry of Education, MOE Key Laboratory of Major Diseases in Children; Hematology Oncology Center Beijing Children’s Hospital, Capital Medical University, National Center for Children’s Health Beijing China; ^2^ Beijing Keyin Technology Company Limited, Beijing Keyin Evergreen Institutes for Medical Research Company limited Beijing China

**Keywords:** dynamic changes, minimal residual disease, neuroblastoma, plasma cell free DNA, therapy response

## Abstract

**Background:**

To improve cure rates for neuroblastoma (NB), it is important and necessary to evaluate therapy response. Our investigation focuses on using plasma cell free DNA (cfDNA) as a biomarker to determine tumor burden and minimal residual disease (MRD) of NB patients during chemotherapy.

**Methods:**

Total 58 NB patients were recruited from July 2016 to December 2017. Therapy regime and risk classification were based on COG standard and BCH‐NB‐2007 protocol. RECIST study was used to judge response to therapy at the end of fourth cycle of chemotherapy (CC4) and maintenance stage (MS) respectively. Serial quantifications of cfDNA, NSE, and LDH were examined at four stages, including newly diagnosed, second and CC4, and maintenance.

**Results:**

During early chemotherapy, 65.5% of NB kids responded well. Consistently, cfDNA, NSE, and LDH levels were down‐regulated in NB patients with partial remission (PR) compared to those with stable disease (SD). In both training and predicting sets, the levels of cfDNA were significantly comparable between PR and SD only at CC4 stage. To predict the insufficient response to early chemotherapy, the optimal AUC value of cfDNA was 0.732 and 0.747 in training and predicting sets respectively, with a sensitivity of 63.2% and 80% specificity at 11.59 ng/ml and a sensitivity of 68.4% and 90% specificity at 10.35 ng/ml. At MS, responded NB patients were slightly increased up to 70%. This evaluation was confirmed by further decrease in cfDNA and NSE levels during intermediate chemotherapy in comparison with early stage.

**Conclusion:**

The dynamic change of cfDNA was considered as a surrogate biomarker to evaluate tumor burden and MRD of NB during early and intermediate therapy periods.

## 1 INTRODUCTION

Neuroblastoma (NB) is the most common extracranial solid tumor in children, and is notable for its broad range of clinical behaviors.^1^ Although progress has also been made in the treatment for high‐risk NB, the outcome for patients with this clinical phenotype still remains poor, with long‐term survival rates of 50%.^1^, ^2^, ^3^ This low survival rate is mainly due to tumor relapse or regrowth caused by the activation of chemoresistant minimal residual disease (MRD).^4^, ^5^ To evaluate the therapeutic response and disease status of NB patients, the clinical evaluation of a precise diagnosis of MRD by molecular pathology in NB patients remains to be established.

Plasma cell free DNA (cfDNA) detection has been widely studied in malignant tumors but not popular in pediatric tumors,^6^, ^7^, ^8^, ^9^ such as NB. It is important and necessary to determine whether plasma cfDNA could serve as a biomarker for NB. Based on others reports, it is feasible to introduce plasma cfDNA to evaluate the tumor burden in NB patients.^7^, ^10^, ^11^ Our previous work has demonstrated that cfDNA levels could be considered as tumor burden biomarker in NB.^12^ Present investigation will focus on evaluating cfDNA as a biomarker for monitoring NB patients’ response to early stage and intermediate stage of chemotherapy.

## 2 MATERIALS AND METHODS

### 2.1 Patients

Fifty‐eight eligible patients with newly diagnosed NB were recruited in the Hematology Oncology Center, Beijing Children's Hospital (BCH) between July 2016 and December 2017. Upon enrollment, all NB kids were diagnosed and mostly classified into high risk (HR) or intermediate risk based on the COG (Children's Oncology Group) standard. In addition, all these NB kids did not receive any treatment. The chemotherapy regime was guided by BCH‐2007‐NB. All newly diagnosed NB children would receive chemotherapy before surgery. The first 4 cycles were early chemotherapy. The fourth cycle of chemotherapy (CC4) stage is the first key time window to evaluate the response to chemotherapy before surgery. According to the evaluation result at CC4 stage, surgery operation would be done in majority of NB patients. After surgery, NB patients with low or medium risk would be treated with 2‐3 cycles of chemotherapy. In addition, some NB patients with medium risk would be prescribed with radiotherapy and retinoic acid in case of poor pathological prognosis. NB patients with high risk would be treated with continued chemotherapy, autologous stem cell transplantation, local radiotherapy, and retinoic acid after surgery. The period from the end of CC4 to the beginning maintenance was called the intermediate stage of chemotherapy. Maintenance stage (MS) is the second key time window to evaluate the response to therapy after surgery and postsurgery chemotherapy. At MS, there were 40 NB children who qualified with data requirement.

Two rounds of RECIST (Response Evaluation Criteria in Solid Tumors) were done at CC4 and MS, respectively. According to RECIST score, NB children were divided into complete remission (CR), partial remission (PR), stable disease (SD), and progressive disease (PD). From enrollment to MS, serial cfDNA levels were measured at newly diagnosed, second cycle of chemotherapy, CC4, and MS respectively. Chronologically, former 19 PR and 10 SD patients recruited into training set, and latter 19 PR and 10 SD patients recruited into predicting set.

This research and the BCH‐NB‐2007 protocol were approved by the Beijing Children's Hospital Institutional Ethics Committee (No. 2016‐65). Informed consent was obtained from the parents or guardians of each patient according to the Declaration of Helsinki.

### 2.2 Diagnostics and staging systems

Microscopic examinations of bone marrow aspirates and biopsies were performed to determine the presence of NB cells. Serum tumor markers such as lactate dehydrogenase (LDH) and neuron‐specific enolase (NSE) levels were detected for the diagnosis and monitoring of NB. The defining characteristics of HR‐NB include an age of more than 18 months, metastases (International Neuroblastoma Staging System, INSS)‐IV or *MYCN *gene amplification. The BCH‐NB‐2007‐HR protocol was based on the Hong Kong Pediatric Hematology and Oncology Study Group (HKPHOSG).^13^ HR‐NB treatment regimens include 5–7 cycles of induction chemotherapy and surgery, consolidation therapy with autologous hematopoietic stem‐cell rescue and irradiation, and maintenance therapy to treat minimal residual disease. The BCH‐NB‐2007‐LR, MR protocol based on European intermediate and low risk NB protocol, includes 4–6 cycles of chemotherapy and surgery, increase in local radiotherapy, and maintenance therapy for some MR‐NB with unfavorable histologic types.

### 2.3 Quantification of cfDNA

To calculate the absolute cfDNA levels as previous study described,^12^ plasma samples were separated at 10 000 *g* for 3 min from venous blood with EDTA before DNA purification. DNA was purified from 200 μl plasma and eluted by 50 μl elution buffer using QIAamp DNA Blood Mini Kits (Qiagen, Valencia, CA) according to the manufacturer's instructions. The total amount of plasma DNA was represented by the qPCR result with LINE‐1 (long interspersed nuclear element 1) 79 bp primers. A serially diluted standardized solution of human genomic DNA (Thermo Fisher Scientific, Waltham, MA) was used to create a reference standard curve. The concentration of cfDNA in each sample was calculated according to the standard curve. The qPCR reactions were performed in triplicate, and mean values of the triplicates were used for further analysis. The qPCR reaction mixture was 10 μl and contained 2 μl of the eluted DNA, 1 μl (final concentration 0.2 μm) of each forward and reverse primer of LINE‐1 79, 5 μl of UltraSYBR Mixture (Cwbiotech, Beijing, China), and 1 μl of double‐distilled water. Cycling conditions were 1 min at 95°C, 35 cycles of 95°C for 8 s and 60°C for 15 s. Each plate contained a plasma DNA sample, a negative control (water template), and seven serially diluted standard DNA solutions (10 ng/ul, 5 ng/ul, 1 ng/ul, 0.5 ng/ul, 0.25 ng/ul, 0.0625 ng/ul, 0.015 ng/ul).

### 2.4 Statistics

Statistical analysis was performed in R statistical environment (R‐version 3.4.0) and included Mann‐Whitney *U* tests, Wilcoxon matched‐pairs signed rank test, boxplots, and ROC analysis (Bioconductor ROC package). A *P*‐value lower than 0.05 was considered as statistically significant.

## 3 RESULTS

### 3.1 Clinical characters of NB

From the very beginning, 58 pediatric patients aged 3‐148 months (median, 35 months) with newly diagnosed NB were enrolled. All these NB kids were treated with chemotherapy at least for CC4. As shown in Table 1, children aged from 18 to 60 months accounted for 46.6% of the patients. Male patients were as many as female. Expectedly, 77.6% of primary tumor sites were detected in the retroperitoneal and adrenal region while other locations accounted for only 22.4%, including the thorax and others. In addition, 72.4% NB were classified into HR (high risk) while 10.3% into MR (medium risk), 17.3% into LR (low risk). Approximately, 56.9% of the NB patients had 1 or 2 metastatic sites, while 29.3% had 3 sites. NB with metastasis in more than 3 organs comprised 13.2% of the patients. The most frequent metastatic sites were bone, bone marrow, and distant lymph nodes, which occupied 56.9%, 62.1%, and 43.1%, respectively.

**Table 1:** Demographic and clinical features of enrolled NB (N = 58)

Characteristics	Total cases, N (%)
Age (months)	
<18	20 (34.5)
≥18 and <60	27 (46.6)
≥60	11 (18.9)
Sex	
Female	29 (50.0)
Male	29 (50.0)
Primary site	
Abdomen	45 (77.6)
Thorax	8 (13.8)
Other	5 (8.6)
Tumor size	
≥8 cm	29 (50)
<8 cm	29 (50)
Tumor stage	
II/III	5 (8.6)
IV	53 (91.4)
Risk	
HR	42 (72.4)
MR	6 (10.3)
LR	10 (17.3)
*MYCN* gene	
Amplification	11 (19.0)
Nonamplification	47 (81.0)
Total cfDNA	
≥120 ng/ml	29 (50)
<120 ng/ml	29 (50)
NSE (ng/ml)	
<370	37 (63.8)
≥370	21 (36.2)
LDH (IU/L)	
≤500	27 (46.6)
>500 and <1500	23 (39.6)
≥1500	8 (13.8)
Metastatic site	
Bone	33 (56.9)
Bone marrow	36 (62.1)
Distant lymph node	25 (43.1)
Liver	11 (19.0)
Central nervous system	7 (12.1)
Number of organs with metastasis	
<3	33(56.9)
=3	17(29.3)
>3	8(13.2)
RECIST	
PR	38 (65.5)
SD	20 (34.5)

NB: neuroblastoma, LDH: lactate dehydrogenase, NSE: neuron‐specific enolase, PR: partial remission, SD: stable disease.

According to the clinical risk assessment, it seemed that the majority of NB was severe in clinic. *MYCN* copies were amplified in 19% of patients. By calculating maximum diameter, tumor size distribution was from 1.5 to 21.8 cm and the median size was 7.85 cm. These NB patients’ tumor size distributed evenly in the range of less than 8 cm or more than 8 cm. The median of cfDNA levels was 120 ng/ml. According to the clinical serum test, more NB patients showed a NSE level of below 370 ng/ml (63.8%) or a LDH level of lower than 1500 IU/L (46.6%). After CC4, based on the RECIST score, NB patients with PR (partial remission) accounted for 65.5%, and with SD (stable disease) 34.5%, respectively. In other words, around 65.5% NB patients responded well to early chemotherapy.

### 3.2 cfDNA performance in various response groups of NB during early stage of chemotherapy

During the early stage of chemotherapy, the kinetic cfDNA levels were recorded at three stages, newly diagnosed (ND), chemotherapy cycle 2 (CC2), and CC4. In training set, the significant difference of cfDNA levels was only found at CC4 stage (Table 2). The cfDNA quantification of PR patients (10.6 ng/ml) was significantly lower than that of SD patient (18.2 ng/ml) at CC4 stage (*P* < 0.05, Table 2). At ND and CC2 stages, cfDNA levels of PR patients were lower than that of SD patients, but no significant difference (*P* > 0.05, Table 2). Moreover, the decreased percentage of cfDNA did not present significantly at all three stages (*P* > 0.05, Table 2).

**Table 2:** Plasma cfDNA levels in various response groups of NB during early stage of chemotherapy

Characteristics	Subjects n (PR/SD)	PR (min‐max ng/ml)	SD (min‐max ng/ml)	*P*‐value a PR vs SD
ND	Training (19/10)	69.9 (2.2 to 731.0)	372.1 (8.7 to 2068.9)	0.376
	Predicting (19/10)	115.3 (8.0 to 3550)	270.5 (9.2 to 1448.6)	0.150
CC2	Training (19/10)	19.5 (7.0 to 115.0)	37.0 (10.8 to 202.4)	0.194
	Predicting (19/10)	14.7 (4.7 to 132.9)	28.9 (7.9 to 171.1)	0.228
CC4	Training (19/10)	10.6 (4.6 to 73.4)	18.2 (8.6 to 119.4)	**0.044**
	Predicting (19/10)	8.0 (4.1 to 62.4)	18.0 (7.8 to 142. 9)	**0.031**
ND‐CC2 decreased %	Training (19/10	61.1 (99.6 to 329.9)	72.5 (99.4 to 614.9)	0.800
	Predicting (19/10)	80.8 (99.6 to 614.5)	85.2 (99.5 to 179.0)	0.665
ND‐CC4 decreased %	Training (19/10)	69.9 (99.9 to 221.4)	92.0 (99.0 to 850.1)	0.570
	Predicting (19/10)	90.9 (99.9 to 91.6)	90.1 (99.3 to 16.7)	0.800
CC2‐CC4 decreased %	Training (19/10)	34.9 (94.3 to 6.0)	2.9 (94.2 to 177.9)	0.321
	Predicting (19/10)	28.5 (93.5 to 197.1)	31.8 (94.8 to 112.1).	0.800

*P* < 0.05 was considered as statistically significant (bold).

a presented Mann‐Whitney *U* Test.

In predicting set, cfDNA level of PR patients (8.0 ng/ml) was significantly lower than that of SD patients (18.0 ng/ml) at CC4 stage (*P* < 0.05, Table 2). There was no significant difference between PR and SD groups at ND and CC2 stages (*P* > 0.05, Table 2). In addition, decreased percentage of cfDNA had no significant difference at all three stages (*P* > 0.05, Table 2). The results of both training and predicting sets indicated that the significant down‐regulation of cfDNA in PR group was associated with well response to present chemotherapeutic regime at CC4 stage (the last stage of early chemotherapy).

### 3.3 NSE and LDH performance in various response groups of NB during early stage of chemotherapy

In parallel, two serum biomarkers, NSE and LDH, were measured by blood analysis in NB children at ND, CC2 and CC4. In training set, NSE measurement had no significant difference between PR and SD patients at all three stages (*P* > 0.05, Table 3). The decline of NSE showed no statistical difference at all three stages too (*P* > 0.05, Table 3). In predicting set, the differences of NSE levels was significantly distinct at CC4 stage between PR and SD patients, 19.1 ng/ml vs 25.9 ng/ml, (*P* < 0.05, Table 3). No significant differences were presented at ND and CC2 stages (Table 3). Consistently, the percentage of NSE decline was significantly different between PR and SD patients at CC4 stage (35.3% vs 20%, Table 3), but not at ND and CC2 stages.

**Table 3:** Serum NSE alternation in various response groups of NB during early stage of chemotherapy

Characteristics	Subjects n (PR/SD)	PR (min‐max ng/ml)	SD (min‐max ng/ml)	*P*‐value a PR vs SD
ND	Training (19/10)	233.0 (19.3‐370.0)	269.5 (62.1‐370.0)	0.728
	Predicting (19/10)	221.8 (17.9‐370.0)	370.0 (24.6‐370.0)	0.313
CC2	Training (19/10)	28.1 (13.8‐107.4)	28.7(21.6‐258.0)	0.463
	Predicting (19/10)	26.1 (12.9‐134.9)	36.2 (10.3‐218.0)	0.955
CC4	Training (19/10)	22.0 (10.7‐47.9)	22.4 (13.7‐80.7)	0.396
	Predicting (19/10)	19.1 (8.7‐45.1)	25.9 (15.8‐157.0)	**0.046**
ND‐CC2 decreased %	Training (19/10)	76.7 (28.5‐95.9)	79.4 (30.3‐94.1)	0.925
	Predicting (19/10)	77.9 (−45.8‐93.2)	85.4 (0.0‐97.2)	0.855
ND‐CC4 decreased %	Training (19/10)	87.1 (22.8‐96.3)	87.5 (51.7‐96.3)	0.976
	Predicting (19/10)	87.7 (16.8‐96.9)	85.6 (−7.3‐95.7)	0.392
CC2‐CC4 decreased %	Training (19/10)	31.4 (−27.5‐63.5)	31.0 (−20.8‐83.5)	0.977
	Predicting (19/10)	35.3 (−130.1‐85.5)	20.7 (−169.9‐59.3)	**0.049**

*P* < 0.05 was considered as statistically significant (bold).

a presented Mann‐Whitney *U* Test.

Unlike NSE, LDH levels had no significant difference between PR and SD patients at any stages (Table 4). The LDH performance suggested that LDH could not monitor therapy response during early chemotherapy stage.

**Table 4:** Serum LDH alternation in various response groups of NB during stage of chemotherapy

Characteristics	Subjects n (PR/SD)	PR (min‐max IU/L)	SD (min‐max IU/L)	*P*‐Value^a^ PR vs SD
ND	Training (19/10)	497 (177‐1893)	568.5 (301‐5540)	0.454
	Predicting (19/10)	447 (251‐3738)	575.5 (254‐2894)	0.396
CC2	Training (19/10)	335 (163‐524)	367(186‐1279)	0.207
	Predicting (19/10)	358 (200‐646)	359 (171‐663)	0.925
CC4	Training (19/10)	264 (164‐417	332 (169‐440)	0.282
	Predicting (19/10)	277 (163‐667)	284.5 (200‐559)	0.884
ND‐CC2 decreased %	Training (19/10)	44.8 (−33.3‐88.0)	33.0 (−5.3‐94.3)	0.855
	Predicting (19/10)	33.1 (−154.3‐89.9)	26.1 (−31.1‐90.0)	0.732
ND‐CC4 decreased %	Training (19/10)	50.0 (−21.6‐89.4)	45.1 (−22.6‐95.9)	0.941
	Predicting (19/10)	28.7 (−122.7‐94.2)	55.5 (6.0‐92.6)	0.376
CC2‐CC4 decreased %	Training (19/10)	9.0 (−49.7‐55.2)	23.0 (−34.7‐68.3)	0.329
	Predicting (19/10)	13.0 (−156.4‐61.3)	21.8 (−43.3‐44.4)	0.812

a presented Mann‐Whitney *U* Test.

### 3.4 Performance of cfDNA and NSE levels for predicting insufficient response to chemotherapy

**Figure 1:**
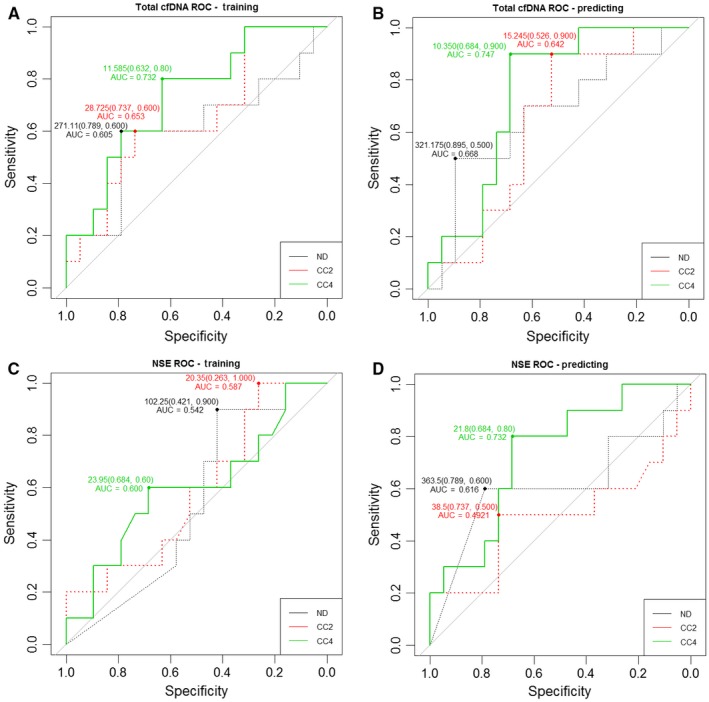
Receiver‐operating curves (ROC) for predicting insufficient response to early chemotherapy in NB patients by cfDNA and NSE levels. The area under the ROC curve (AUC) of total plasma cfDNA was analyzed both in training set (A) and predicting set (B), respectively. The AUC of NSE was analyzed both in training set (C) and predicting set (D), respectively

The performance of cfDNA at CC4 stage showed consistent difference between PR and SD patients in both training and predicting sets (Tables 2), while NSE examination only performed well at CC4 stage in predicting set (Table 3). Next, ROC (Receiver operation curve) curves were plotted to test whether the two factors had potential to predict insufficient response to chemotherapy during early stage. In training set, the ROC analysis of cfDNA levels at all three stages was able to predict insufficient response (SD) to chemotherapy with a similar sensitivity and specificity (Figure 1A). The optimal AUC (area under ROC curve) of cfDNA was 0.732 with a sensitivity of 63.2% at 80.0% specificity at 11.585 ng/ml at CC4 stages, (Figure 1B). In predicting set, the optimal AUC of cfDNA was 0.747 with a sensitivity of 68.4% at 90.0% specificity at 10.35 ng/ml at CC4 (Figure 1B). As for NSE, the optimal AUC was 0.732 at cutoff value of 21.8 ng/ml with a sensitivity of 68.4% at 80.0% specificity at CC4 stage in predicting set (Figure 1C,D).

### 3.5 cfDNA, NSE, and LDH performance in maintenance stage

**Figure 2:**
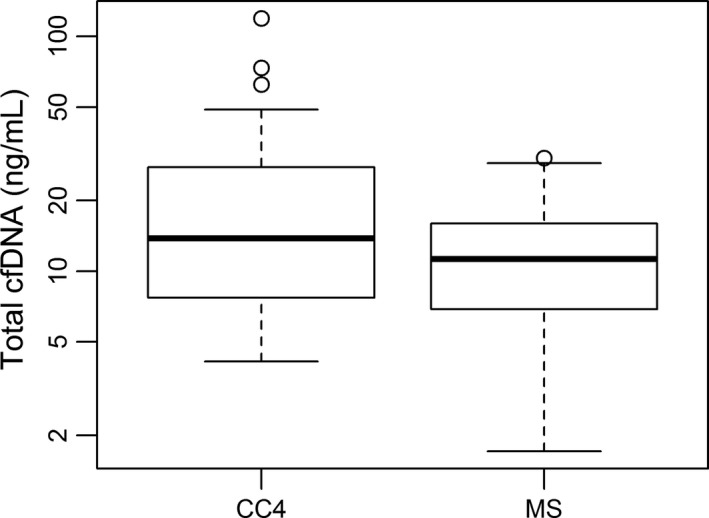
Comparison of cfDNA level between NB patients at CC4 and MS (maintenance stage). Box plots for comparison of cfDNA levels in NB patients between CC4 (13.80 ng/ml) and MS (11.25 ng/ml) was significant (*P* = 0.02, <0.05). (Wilcoxon matched‐pairs signed rank test)

**Figure 3:**
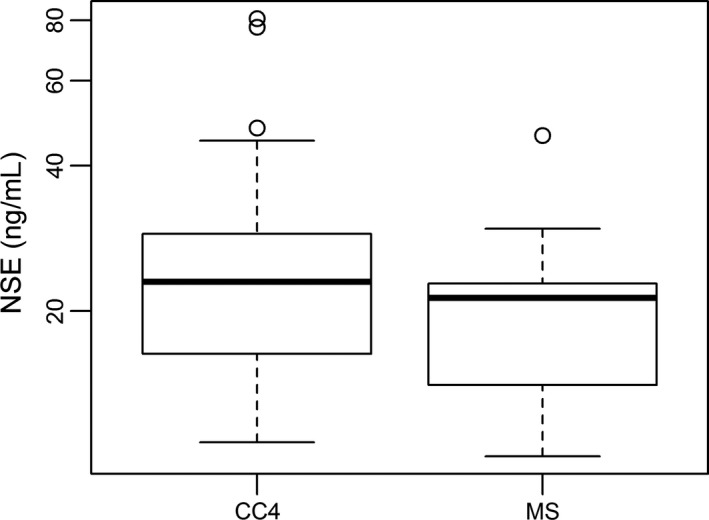
Comparison of NSE level between NB patients at CC4 and MS (maintenance stage). Box plots for comparison of NSE levels in NB patients between CC4 (24.0 ng/ml) and MS (21.30 ng/ml) was significant (*P* = 0.01, <0.05). (Wilcoxon matched‐pairs signed rank test)

**Figure 4:**
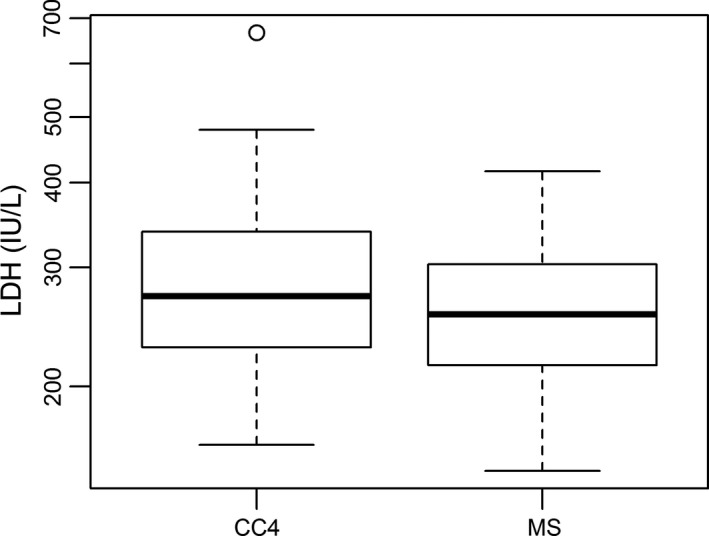
Comparison of LDH level between NB patients at CC4 and MS (maintenance stage). Box plots for comparison of LDH levels in NB patients between CC4 (272.0 IU/L) and MS (255.5 IU/Ll) was not significant (*P* = 0.11, >0.05). (Wilcoxon matched‐pairs signed rank test)

To some extent, cfDNA levels could be distinguished from response and insufficient response during the early stage of chemotherapy in NB patients. Furthermore, our study recorded cfDNA levels of NB kids at the MS. At this time window, all NB patients were re‐scored based on RECIST. Very interestingly, the well responded NB patients were 70% (Table 5), a slightly higher than it at CC4 stage. As expected, all three factors, including cfDNA, NSE and LDH, had no significant differences between PR and SD groups (Table 6). Furthermore, the statistical difference of cfDNA levels was analyzed between CC4 and MS. Under expectation, the level of cfDNA in MS (11.25 ng/ml) was significantly lower than it in CC4 (13.80 ng/ml) (*P* = 0.0246, <0.05, Figure 2). At the same time, NSE level was significantly down‐regulated in MS in comparison to CC4, 21.3 ng/ml vs 24 ng/ml, (*P* = 0.0116, <0.05), (Figure 3). But LDH level did not show significant changes between NB patients at MS and CC4 stages, 255.5 IU/L vs 272 IU/L, *P* = 0.1528 (>0.05), (Figure 4).

**Table 5:** Demographic and clinical features of patients at maintenance stage (N = 40)

Characteristics	Total cases, N (%)
Age (months)	
<18	13 (32.5)
≥18 and <60	21 (52.5)
≥60	6 (15)
Sex	
Female	19 (47.5)
Male	21 (52.5)
RECIST	
PR	28 (70.0)
SD	12 (30.0)

**Table 6:** Blood biomarkers performance in NB with maintenance stage (N = 40)

	PR	SD	*P*‐value^a^
Subjects n	28	12	
cfDNA ng/mL	11.25(1.71‐30.35)	11.4(3.65‐28.81)	0.871
CC4‐MS % decrease	3.45(−206.13‐89.01)	52.25(−88.13‐88.43)	0.1361
NSE ng/ml	20.55(10.4‐46.2)	21.8(10.0‐25.1)	0.7231
LDH IU/L	270(150‐359)	219.5(162‐416)	0.262

a Mann‐Whitney U Test.

**Figure 5:**
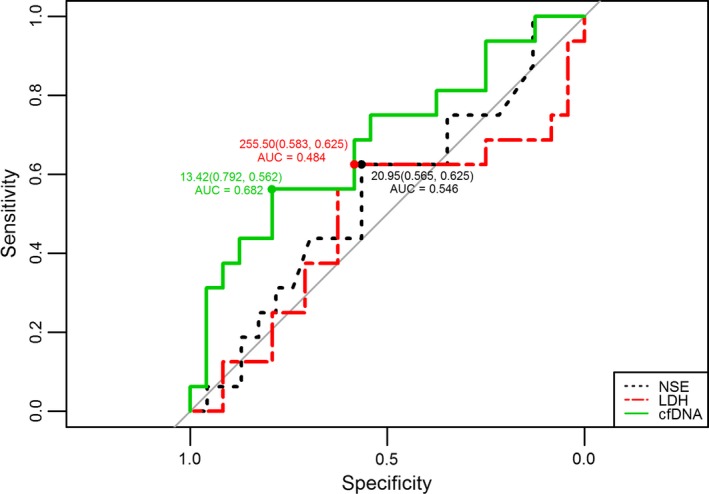
Receiver‐operating curves (ROC) for predicting insufficient response to intermediate chemotherapy in NB patients by cfDNA, NSE, and LDH levels. The area under the ROC curve (AUC) of total plasma cfDNA was 0.682 (with 79.2% sensitivity and 56.2% specificity at 13.42 ng/ml) at maintenance stage (MS), NSE was 0.546 (with 56.5% sensitivity and 62.5% specificity at 20.95 ng/ml), and LDH was 0.484 (with 58.3% sensitivity and 62.5% specificity at 255.50 IU/L, respectively

In addition, to predict the insufficient response to intermediate stage of chemotherapy, the most efficiency of AUC was found in cfDNA levels among cfDNA, NSE, and LDH quantification (Figure 4). The AUC value of cfDNA was 0.68 at cutoff value of 13.4 ng/ml with a sensitivity of 79.2% at 56.2% specificity (Figure 5). The results of cfDNA and NSE matched with clinical characters. At MS, all NB patients had done with the surgery operation. Consequently, all these NB kids suffering from reduced tumor burden or minimal residual disease had lower cfDNA levels at MS stage than it at CC4 or presurgery stage. However, cfDNA levels could not be able to discriminate PR from SD among NB patients (Table 6).

## 4 DISCUSSION

To improve cure rates of malignant tumors, such as NB, therapy response is the key factor to be considered.^14^, ^15^ Meanwhile, MRD detection plays a more pivotal role in predicting prognosis.^2^, ^14^, ^15^, ^16^, ^17^, ^18^ When it comes to NB, particularly the NB with high risk, their outcomes are usually negative due to tumor relapse or metastasis.^1^, ^2^, ^14^ Although *MYCN* and MIBG had some advantages to detect MRD for NB,^11^, ^19^, ^20^ disappointedly, none of them were good or sensitive enough to determine MRD. Physicians are eager to find a number of reliable biomarkers which can measure tumor burden or MRD of NB easily and accurately.

Our previous work has demonstrated that cfDNA levels could be considered as one type of promising biomarker of tumor burden in NB.^12^ Present investigation focused on evaluating cfDNA as a biomarker for monitoring response to early and intermediate stages of chemotherapy in NB. Similar to our previous finding, the cfDNA levels were gradually down‐regulated following therapy process (Table 2, Figure 2). More importantly, cfDNA levels could be used to discriminate PR from SD during early chemotherapeutic stage (Table 2). To some degree, using cfDNA could predict the insufficient response to early chemotherapy in NB (Figure 1A,B). Higher level of cfDNA was found in SD patients (Table 2, Figure 1A,B). In comparison with NSE and LDH performance at both early and intermediate stages of chemotherapy, the performance of cfDNA was most efficient to evaluate therapy response in NB patients (Tables 2, 3, 4, Figures 1, 2, 3, 4, 5). Our data proved that cfDNA levels were sensitively down‐regulated upon treatment, such as surgery or chemotherapy (Tables 2 and 6). This correlation between cfDNA level and tumor burden could facilitate predicting therapy response more efficiently.

However, there were some factors to affect cfDNA's stability and efficiency. For instance, 14 eligible NB patients’ cfDNA levels were too fluctuant to reveal the significant difference between PR and SD groups during early chemotherapy (data not shown). By dissecting the clinical characters, three factors seemed to stimulate cfDNA up‐regulating suddenly, including inflammation fever,^22^ transfusion,^23^ and administration of G‐CSF.^24^ As a result, the suddenly increased cfDNA levels disturbed our investigation. By re‐sampling from the 14 NB kids in case of the three disturbing factors disappearing, the real tumor burden was measured by cfDNA and the real response to therapy was analyzed. Then, it was necessary to avoid the time windows when disturbing factors were involved during detecting cfDNA.

Given that precise tumor burden or MRD could be measured dynamically, it would benefit not only the cancer patients but also the clinicians in clinic. At present, imaging technique is the best and most widely used method to facilitate diagnosis and prognosis for malignant solid tumors.^20^, ^25^, ^26^ However, radiography has some drawbacks, for example it has radiation danger and it is also challenging to detect minimum tumor size.^20^, ^25^ Getting this notion, the research of discovering safer and more dynamic biomarkers for malignant tumors are emerging as frontier topic both in basic and clinic studies,^6^, ^7^, ^10^, ^11^, ^16^, ^17^ and this includes cfDNA quantification. Recently, it was demonstrated that the fragment size of cfDNA was reported to improve sensitivity and specificity of various cancers test.^27^ Actually, our previous work had detected two fragment lengths of cfDNA in NB patients. The smaller fragment size (97 base pairs) of cfDNA had more significant performance than the DNA integrity index (longer over shorter fragments).^12^ Furthermore, optimizing works would focus on more specific fragment size of cfDNA to evaluate tumor burden of NB.

In conclusion, our investigation revealed that cfDNA levels were gradually down‐regulated in response to early and intermediate stages of chemotherapy in NB. More importantly, dynamic cfDNA changes could be used to monitor and predict therapy response at CC4 and MS stages in NB. If larger samples were available, cfDNA level could be applied in a wider and more accurate scenario to evaluate and predict therapy response as well as follow‐up prognosis in NB.

## CONFLICTS OF INTEREST

The authors declare that there are no conflicts of interest.
